# A recombinant spike‐XBB.1.5 protein vaccine induces broad‐spectrum immune responses against XBB.1.5‐included Omicron variants of SARS‐CoV‐2

**DOI:** 10.1002/mco2.263

**Published:** 2023-04-26

**Authors:** Cai He, Aqu Alu, Hong Lei, Jingyun Yang, Weiqi Hong, Xiangrong Song, Jiong Li, Li Yang, Wei Wang, Guobo Shen, Guangwen Lu, Xiawei Wei

**Affiliations:** ^1^ Laboratory of Aging Research and Cancer Drug Target State Key Laboratory of Biotherapy and Cancer Center National Clinical Research Center for Geriatrics West China Hospital Sichuan University Chengdu China

**Keywords:** neutralizing antibody, Omicron, RBD mutations, recombinant protein vaccine, SARS‐CoV‐2, XBB.1.5

## Abstract

The XBB.1.5 subvariant has drawn great attention owing to its exceptionality in immune evasion and transmissibility. Therefore, it is essential to develop a universally protective coronavirus disease 2019 vaccine against various strains of Omicron, especially XBB.1.5. In this study, we evaluated and compared the immune responses induced by six different spike protein vaccines targeting the ancestral or various Omicron strains of severe acute respiratory syndrome coronavirus 2 (SARS‐CoV‐2) in mice. We found that spike‐wild‐type immunization induced high titers of neutralizing antibodies (NAbs) against ancestral SARS‐CoV‐2. However, its activity in neutralizing Omicron subvariants decreased sharply as the number of mutations in receptor‐binding domain (RBD) of these viruses increased. Spike‐BA.5, spike‐BF.7, and spike‐BQ.1.1 vaccines induced strong NAbs against BA.5, BF.7, BQ.1, and BQ.1.1 viruses but were poor in protecting against XBB and XBB.1.5, which have more RBD mutations. In sharp contrast, spike‐XBB.1.5 vaccination can activate strong and broadly protective immune responses against XBB.1.5 and other common subvariants of Omicron. By performing correlation analysis, we found that the NAbs titers were negatively correlated with the number of RBD mutations in the Omicron subvariants. Vaccines with more RBD mutations can effectively overcome the immune resistance caused by the accumulation of RBD mutations, making spike‐XBB.1.5 the most promising vaccine candidate against universal Omicron variants.

## 1 INTRODUCTION

Since 2020, severe acute respiratory syndrome coronavirus 2 (SARS‐CoV‐2) has undergone continuous evolution from its ancestral WA1/2020 strain to adapt to humans. Various variants of concern (VOCs) emerged gradually from Alpha (B.1.1.7), Beta (B.1.351), Gamma (P.1), Delta (B.1.617.2), and Omicron (B.1.1.529) variants, becoming dominant regionally or globally at different times.^1^ Among these VOCs, Omicron is the most heavily mutated one with high transmissibility and resistance to antiviral immunity.^2^ Since the discovery of the first Omicron (B.1.1.529) in South Africa in November 2021, novel sublineages, subvariants, and recombinant strains have emerged constantly starting from the earliest BA.1 strain to the latest XBB.1.5 strain, resulting in new waves of pandemics.^2^, ^3^, ^4^ Five lineages of Omicron variants have been identified to date, including BA.1, BA.2, BA.3, BA.4, and BA.5, three of which (BA.1, BA.2, and BA.5) have become dominant worldwide.^5^ Since January 2023, the incidence of Omicron BQ.1 with its sublineages BQ.1.1 (derived from BA.5 subvariant) and XBB (a recombination of two BA.2 subvariants) is in rapid elevation in different countries, replacing previously dominating strains such as BA.5.^6^, ^7^ BQ.1.1 and XBB sublineages contain mutations in the receptor‐binding domain (RBD) of spike protein, which is the major target for vaccine design and immunotherapy for SARS‐CoV‐2.^8^ These lineages carry more mutations in RBD than their ancestor BA.5 and BA.2 strains, leading to enhanced immune evasion and transmissibility.^9^ Previous studies indicated that BQ.1.1 and XBB showed higher resistance to the humoral immunity induced by vaccination, natural infection, or therapeutic monoclonal antibodies than earlier Omicron strains such as BA.2 and BA.5.^8^, ^10^

XBB.1.5 is a descendant of XBB and has been considered the most efficient and contagious strain so far. Since its first discovery in late 2022, it has experienced high transmissibility and rapid expansion, outcompeted BQ.1.1, and became the dominant lineage in the USA, making up an estimated 89.6% of new cases in the latest reports.^11^ Among different Omicron subvariants, XBB.1.5 possesses the most mutations in the RBD, amounting up to 22 when compared with the RBD of the ancestral SARS‐CoV‐2. These mutations include G339H, R346T, F486P, F490S, etc.^12^ XBB.1.5 and XBB share most of the amino acid substitutions in the RBD. However, the additional F486P substitution in XBB.1.5 endows its substantially higher affinity to human angiotensin‐converting enzyme 2 (ACE2) receptors than BQ.1, XBB, and XBB.1.^13^, ^14^ The dissociation constant value of RBD from human ACE2 receptors is 4.3 times lower for XBB.1.5 when compared to XBB.1 subvariant.^13^ This unique F486P mutation provided XBB.1.5 with superior infection and growth advantages over other Omicron variants. Besides, XBB.1.5 is also profoundly immunotolerant to humoral immunity induced by coronavirus disease 2019 (COVID‐19) vaccines (both monovalent and bivalent), commercial monoclonal antibodies, and convalescent plasma treatment.^13^, ^15^, ^16^, ^17^ It is the most resistant SARS‐CoV‐2 variant ever. Considering the high transmissibility and remarkable immune evasion, XBB.1.5 would probably become the cause of the next global wave. Therefore, it is essential to develop next‐generation and updated COVID‐19 vaccines targeting XBB sublineages, especially XBB.1.5, to provide better protection and pandemic control in advance.

To alleviate the Omicron pandemic, a bivalent vaccine that targets both the wild‐type (WT, D614G) and BA.4–BA.5 spike proteins of SARS‐CoV‐2 (Pfizer–BioNTech) has been approved for emergency use in many countries. Compared with the original BNT162b2 monovalent vaccine, a fourth booster dose of the bivalent vaccine induced higher neutralizing responses against a bunch of Omicron sublineages.^18^, ^19^ However, the neutralizing titers against BA.2.75.2, BQ.1.1, and XBB.1 exhibited a sharp decrease compared with BA.4–BA.5 strain after the bivalent vaccine.^16^, ^18^ In addition, the bivalent vaccine may have some efficacy against BQ.1.1, it largely fails to protect against XBB.1 and XBB.1.5 infection with the neutralizing antibody (NAb) titers remaining below the detection threshold in 32%−42% individuals.^16^ Meanwhile, the relatively low NAb titers against XBB.1 and XBB.1.5 decreased more quickly to baseline levels before boosting the bivalent vaccine than other Omicron variants.^20^ Thus, people who have built up anti‐COVID‐19 immunity via previous infection or vaccination are at high risk of breakthrough infection with XBB‐related lineages again. Although no evidence has indicated that XBB.1.5 is more dangerous than other strains, XBB.1.5‐related hospitalizations and deaths may still elevate since an increasing number of people overall will be infected and reinfected. As XBB.1.5 is highly resistant to antiviral immunotherapy, the most efficient way to control the upcoming wave is to develop vaccines that can induce effective immunity against XBB.1.5.

Among different types of COVID‐19 vaccines, recombinant protein vaccines show superiority in safety and easiness to manufacture. We have developed several protein‐based COVID‐19 vaccines that induced strong immunity against the ancestral SARS‐CoV‐2 and some Omicron variants.^21^, ^22^, ^23^ In this study, we evaluated the immunogenicity of various protein‐based COVID‐19 vaccines that target the spike proteins of the WT and different Omicron variant strains (BA.5, BF.7, BQ.1.1, XBB, and XBB.1.5). We discovered that among different vaccines tested, spike‐XBB.1.5 vaccine induced the most broadly protective immunity against a wide range of Omicron subvariants, especially the most contagious XBB.1.5. Meanwhile, by performing correlation analysis, we discovered a close relationship between the number of mutations in RBD and NAb titers for the first time. We aimed to develop a universal vaccine against SARS‐CoV‐2 Omicron and inspire the production of next‐generation vaccines for future variants of SARS‐CoV‐2.

## 2 RESULTS

### 2.1 Evaluation of the immunogenicity of COVID‐19 vaccines targeting various Omicron variants

**FIGURE 1:**
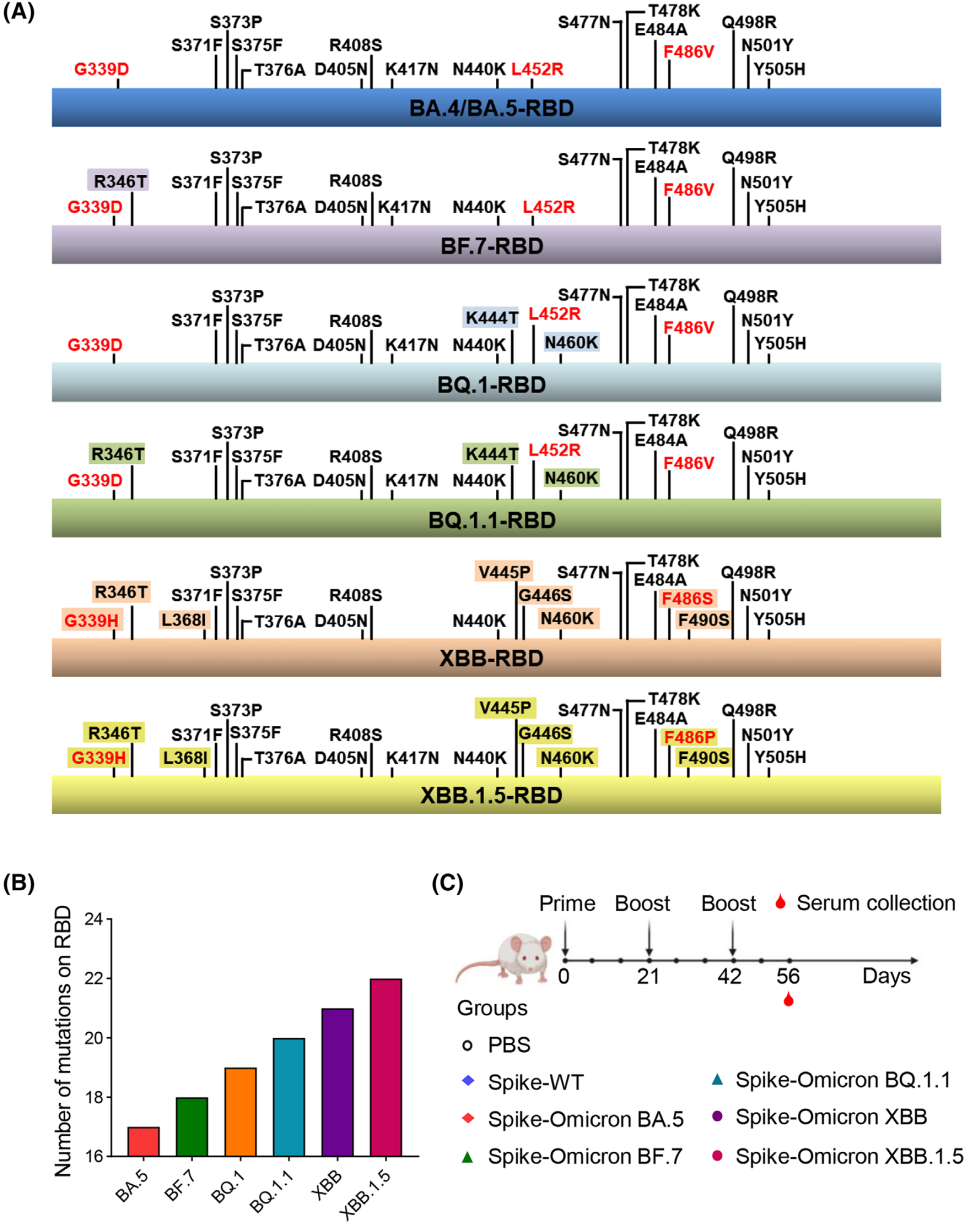
(A) Schematic illustration of the mutations in receptor‐binding domain (RBD) proteins of various Omicron subvariants. (B) Numbers of mutations in the RBD of various Omicron subvariants. (C) Scheme of immunization. Mice (*n* = 6/group) were immunized with PBS, spike‐WT, spike‐Omicron BQ.1.1, spike‐Omicron BA.5, spike‐Omicron XBB, spike‐Omicron BF.7, or spike‐Omicron XBB.1.5 on days 0, 21, and 42 intramuscularly. On day 56, serum samples were collected for further studies.

The mutations in the RBD of the main circulating Omicron sublineages at present are summarized in Figure 1A, including BA.5, BF.7, BQ.1, BQ.1.1, XBB, and XBB.1.5. As shown in Figure 1B, the number of mutations in RBD varied in different Omicron subvariants when compared with the ancestral RBD of SARS‐CoV‐2, with BA.5 carrying the least (17) and XBB.1.5 carrying the most (22) mutations. XBB and XBB.1.5 share most of the amino acid substitutions in the RBD except for additional K417N and F486P substitutions in XBB.1.5. Similarly, BA.5 and BF.7 have many mutations in common except for an additional R346T mutation in BF.7, which are co‐circulating strains in the current outbreak in China owing to the relaxation of the dynamic zero‐COVID policy.^24^, ^25^

**FIGURE 2:**
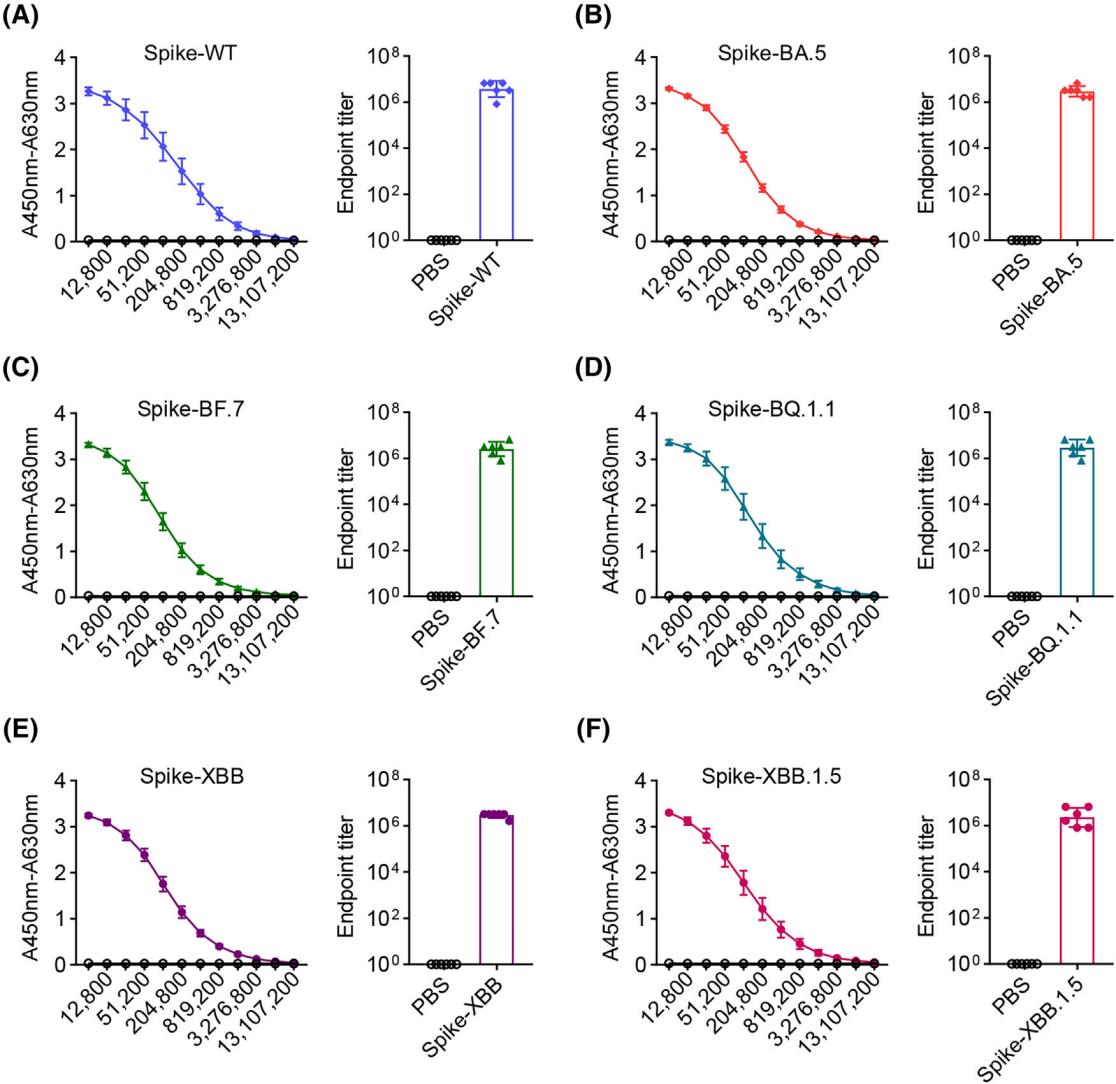
Immunization with recombinant spike protein vaccines induced strong immunoglobulin G (IgG) responses. Detection of spike‐WT (A), spike‐BA.5 (B), spike‐BF.7 (C), spike‐BQ.1.1 (D), spike‐XBB (E), and spike‐XBB.1.5 (F)‐specific IgG antibody titers in the serum of mice immunized with corresponding vaccines. Left: absorbance values at 450 nm indicating the binding to serially diluted antibodies; right: endpoint titers of IgG antibodies in the sera of vaccinated animals (*n* = 6). Data are presented as geometric mean values ± SD.

To evaluate the humoral immune responses, NIH mice (*n* = 6 per group) were immunized intramuscularly with phosphate buffer solution (PBS) or six spike proteins targeting WT SARS‐CoV‐2 or various Omicron subvariants on days 0, 21, and 42, including spike‐WT, spike‐BA.5, spike‐BF.7, spike‐BQ.1.1, spike‐XBB, and spike‐XBB.1.5 (Figure 1C). Two weeks after the last immunization, mouse sera were collected to evaluate both antigen‐specific and cross‐protective antibody responses. The WT spike or spike proteins with specific mutations were used as coated antigens to determine the total immunoglobulin G (IgG) antibody titers. As shown in Figure 2A, three doses of spike‐WT induced high levels of serum IgG antibodies targeting the unmutated spike protein, with an endpoint titer of 3,678,084. Enzyme‐linked immunosorbent assay (ELISA) analysis with spike‐BA.5 protein indicated that the spike‐BA.5 vaccine also showed comparable capability in inducing serum antigen‐specific IgG responses (2,919,297) (Figure 2B). Similar results were observed in the spike‐BF.7 (Figure 2C), spike‐BQ.1.1 (Figure 2D), spike‐XBB (Figure 2E), and spike‐XBB.1.5 (Figure 2F) groups. These results suggested that mutations in spike proteins did not compromise the immunogenicity of the spike proteins. All of the prototype and mutated spike proteins induced high levels of antigen‐specific humoral responses in mouse sera after three‐dose vaccination.

### 2.2 Spike‐XBB.1.5 vaccine induced broad neutralization of Omicron variants

**FIGURE 3:**
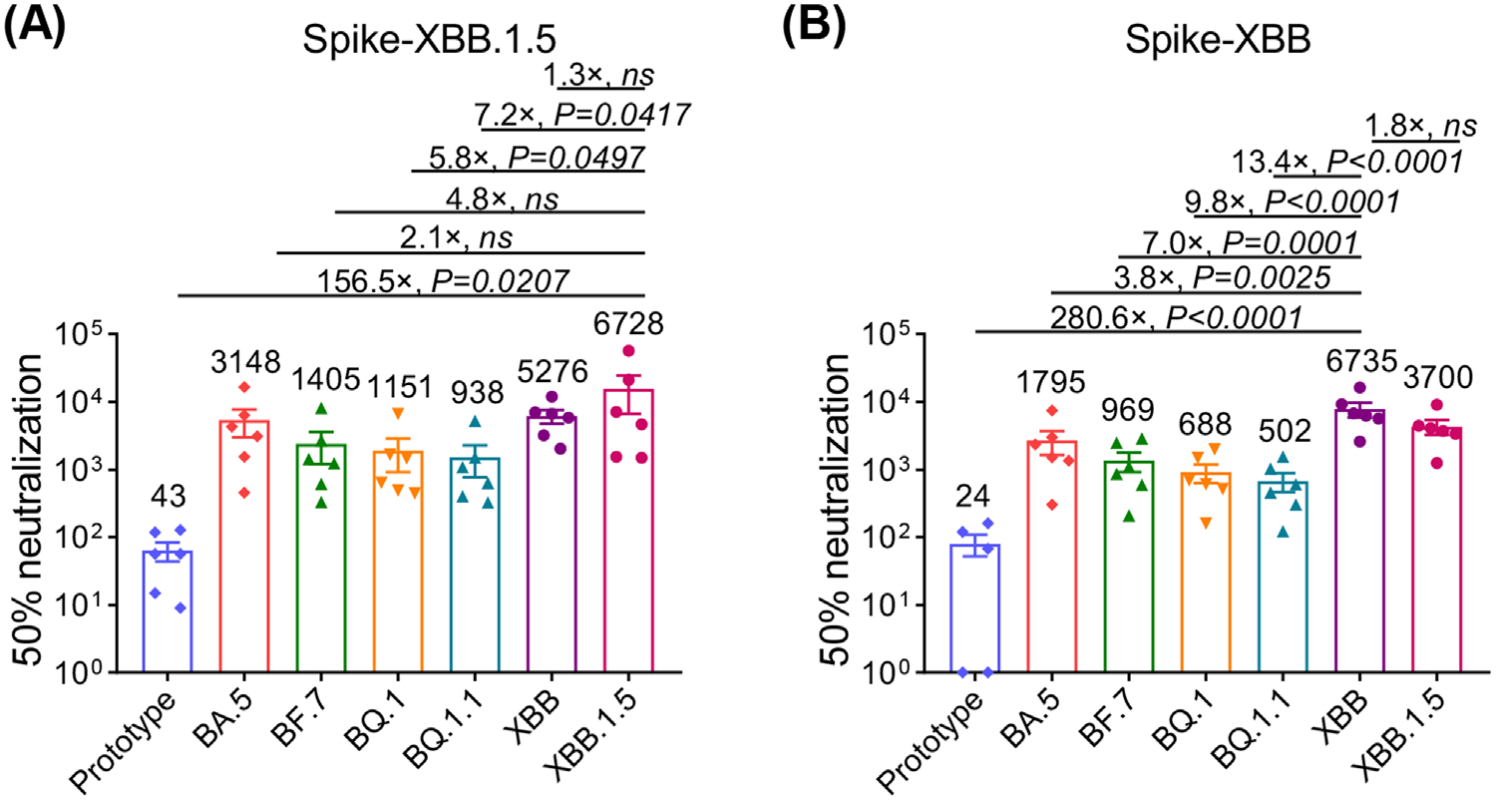
Immune sera from mice immunized with spike‐XBB and spike‐XBB.1.5 neutralized the infection of multiple Omicron variants. Fifty percent neutralizing titers of immune sera from mice immunized with spike‐XBB.1.5 (A) or spike‐XBB (B) against luciferase‐expressing pseudoviruses of the WT strain or various Omicron subvariants, including BA.5, BF.7, BQ.1, BQ.1.1, XBB, and XBB.1.5. Fifty percent neutralization is defined as the dilution titer that neutralized 50% infection (*n* = 6). Data are presented as geometric mean values ± SD.

To determine the functions of the antibodies, we next investigated the neutralizing capacities of the immune sera against the prototype or mutated SARS‐CoV‐2. Pseudoviruses of WT, BA.5, BF.7, BQ.1, BQ.1.1, XBB, and XBB.1.5 were used with a luciferase assay, which can simulate the infection and entry of authentic virus with attenuated virulence.^26^ We found that immunization with spike‐XBB.1.5 induced high titers of antigen‐specific NAbs, with a 50% neutralizing titer of 6728 (Figure 3A). Spike‐XBB.1.5 immunization also showed high protectivity against other Omicron subvariants. Despite a slight downward trend, no significant change was observed in the protective effect of immune sera from spike‐XBB.1.5‐immunized mice on XBB, BA.5, and BF.7 strains. Spike‐XBB.1.5‐induced humoral immunity was also effective against BQ.1 and BQ.1.1 strains, with the 50% neutralization titers dropping by no more than 7.2 times. Therefore, the spike‐XBB.1.5 vaccine could induce strong humoral responses that could not only protect against XBB and XBB.1.5 but also against other Omicron sublineages that are still circulating in different regions. Strikingly, we discovered that the NAb titers against the Omicron subvariants earlier than the XBB strain showed a declining trend as the number of mutations in RBD increased. For instance, the 50% NAb titers were highest against BA.5 (3148) but lowest against BQ.1.1 (938), the latter of which contains three more mutations in the RBD than the former. Unlike the results of the Omicron variants, the spike‐XBB.1.5 vaccine exhibited a 156.5‐fold decrease in protection against WT pseudovirus infection in 293T/ACE2 cells, with a nearly undetectable 50% NAb titer (43). This may be because, as the virus evolved, there were enough mutations in the RBD of XBB.1.5 to completely replace the antigenic sites in the RBD of the ancestral SARS‐CoV‐2, resulting in the low affinity between WT RBD and the antibodies induced by spike‐XBB.1.5. As the current COVID‐19 pandemic is dominated by Omicron variants rather than the original strain,^27^, ^28^ spike‐XBB.1.5 could be a promising vaccine candidate against the current dominating strains, including XBB.1.5 subvariant that is mainly responsible for the surge in new infections.

Similar results were observed with the spike‐XBB vaccine. When immunized with spike‐XBB, the immune sera showed minimal efficacy in neutralizing the infection of the prototype SARS‐CoV‐2 (Figure 3B). As expected, spike‐XBB protein‐induced antibodies showed superior protection against XBB pseudovirus. Meanwhile, the immune sera exhibited favorable efficacy against XBB.1.5 with a 50% neutralizing titer of 3700. However, a 3.8–13.4‐fold reduction in the neutralizing capacity was observed when the immune sera were collected to prevent the infection of other Omicron strains in 293T/ACE2 cells, including BA.5, BF.7, BQ.1, and BQ.1.1. When spike‐XBB's immune sera were used to neutralize subvariants with more RBD mutations, a downward trend was also observed in 50% NAb titers, from 1795 for BA.5 to 502 for BQ.1.1. These results indicated that spike‐XBB and spike‐XBB.1.5 protein vaccines, which have relatively more mutations in RBD, are highly effective against XBB, XBB.1.5 and earlier Omicron strains. Moreover, since the spike‐XBB.1.5 vaccine has one more mutation in RBD than spike‐XBB, it has better protection against Omicron sublineages from BA.5 to BQ.1.1 with approximately double the 50% NAb titer.

### 2.3 Spike‐WT, spike‐BA.5, spike‐BF.7, spike‐BQ.1.1 were poor in protecting against the infection of XBB and XBB.1.5 sublineages

**FIGURE 4:**
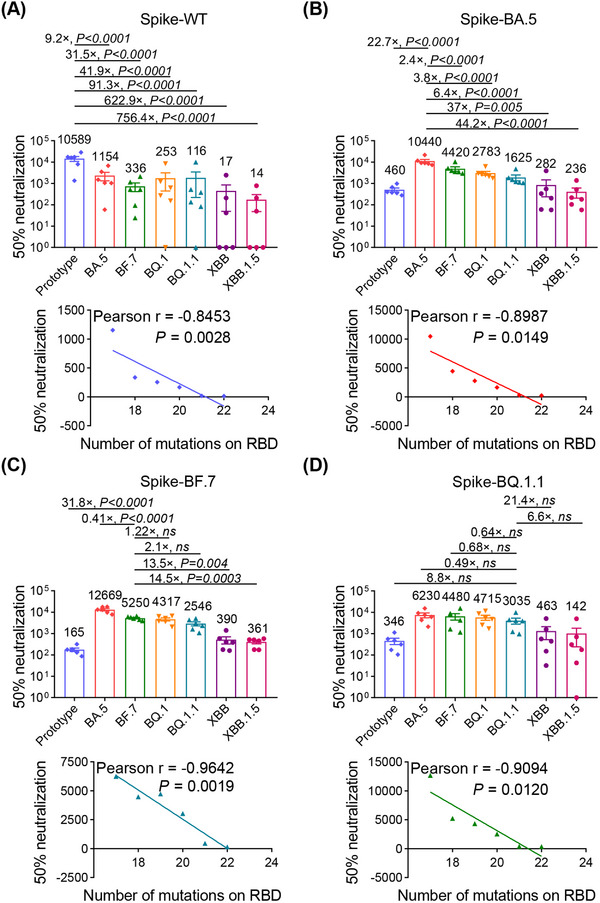
Immune sera from mice immunized with spike‐WT, spike‐BA.5, spike‐BF.7, or spike‐BQ.1.1 showed limited efficacy in neutralizing XBB and XBB.1.5 subvariants. Upper: 50% neutralizing titers of immune sera from mice immunized with spike‐WT (A), spike‐BA.5 (B), spike‐BF.7 (C), or spike‐BQ.1.1 (D) against luciferase‐expressing pseudoviruses of the WT strain or various Omicron subvariants, including BA.5, BF.7, BQ.1, BQ.1.1, XBB, and XBB.1.5. Fifty percent neutralization is defined as the dilution titer that neutralized 50% infection. Lower: Pearson correlation analysis between 50% neutralizing antibody titers and mutation numbers of RBD in Omicron sublineages (*n* = 6). Data are presented as geometric mean values ± SD.

We also characterized the functions of antibodies induced by the other four vaccines with a neutralizing assay. In contrast to spike‐XBB.1.5 and spike‐XBB, spike‐WT immunization only induced protective antibodies against the prototype pseudovirus. The overall neutralizing capacity toward BA.5, BF.7, BQ.1, and BQ.1.1 strains was reduced by 9.2, 31.5, 41.9, and 91.3 times after the spike‐WT vaccine compared to the ancestral SARS‐CoV‐2 (Figure 4A). We also found that XBB and XBB.1.5 subvariants showed almost complete tolerance to the spike‐WT vaccine since the neutralizing activities against XBB (17) and XBB.1.5 (14) were strikingly lower than those against the WT strain (10,589). We next investigated the relationship between NAb titers and RBD mutations within the Omicron sublineages with correlation analysis and discovered a strong negative correlation (Pearson *r* = −0.8453), which means that the efficacy of spike‐WT‐immunized sera in neutralizing Omicron subvariants declines with the addition of mutations in the RBD of the virus. Therefore, the Omicron subvariants demonstrated enhanced capability to evade NAbs induced by spike‐WT vaccine in a mutation‐dependent manner, weakening the value of the spike‐WT in controlling the current SARS‐CoV‐2 pandemic dominated by Omicron variants.

As control, mice were immunized with another three vaccines targeting other Omicron sublineages, including spike‐BA.5, spike‐BF.7, and spike‐BQ.1.1. We found that the antibodies induced by these vaccines were poor in protecting the WT SARS‐CoV‐2 as well (Figure 4B–D). However, they exhibited excellent cross‐protection among the four strains: BA.5, BF.7, BQ.1, and BQ.1.1. Interestingly, we found that the immune sera from vaccinated mice were more potent in neutralizing subvariants with fewer mutations in RBD than the targeted virus strain. For instance, the immune sera from mice receiving the spike‐BF.7 vaccine showed a 50% neutralizing titer of only 5250 against BF.7, but it more than doubled its neutralizing titer against BA.5 variant to 12,669, which has one less mutation on the RBD than BF.7. When the immune sera were used to prevent the infection of Omicron sublineages with more RBD mutations, the NAb titers declined as the mutations grew, which also exhibited a markedly inverse correlation (Figure 4B–D). In particular, the protection of these vaccines against the most mutated XBB and XBB.1.5 subvariants was far from satisfactory. The neutralization effect of antibodies produced by spike‐BA.5, spike‐BF.7, and spike‐BQ.1.1 against XBB.1.5 varied between 142 and 361, which was much lower than that induced by spike‐XBB.1.5 (6728) and spike‐XBB vaccine (3700). Therefore, despite the promising use of spike‐BA.5, spike‐BF.7, and spike‐BQ.1.1 vaccines in the current pandemic, their preventive efficacy in the next global wave of XBB.1.5 infection may be severely dampened.

Thus, vaccination with spike‐XBB.1.5 induced robust and broadly protective antibody responses that can not only prevent the infection of currently dominating Omicron strains (BA.5, BF.7, BQ.1, and BQ.1.1) but also the most transmissible SARS‐CoV‐2 strain to date, XBB.1.5. Meanwhile, we found that the NAb titers were strongly and negatively related to the number of mutations in the RBD of viruses, an attractive vaccine target for SARS‐CoV‐2.^29^ Omicron subvariants with more amino acid substitutions on RBD showed the superior capacity to escape from immune sera neutralization. On the contrary, vaccines containing more RBD mutations can induce broader protective immunity since their mutations can cover those in earlier strains. This makes spike‐XBB.1.5, which has the most amino acid mutations in RBD to date, a hopeful vaccine candidate against the current COVID‐19 pandemic caused by multiple Omicron subvariants.

### 2.4 Strong antigen‐specific T‐cell immunity induced by spike‐XBB.1.5 immunization

**FIGURE 5:**
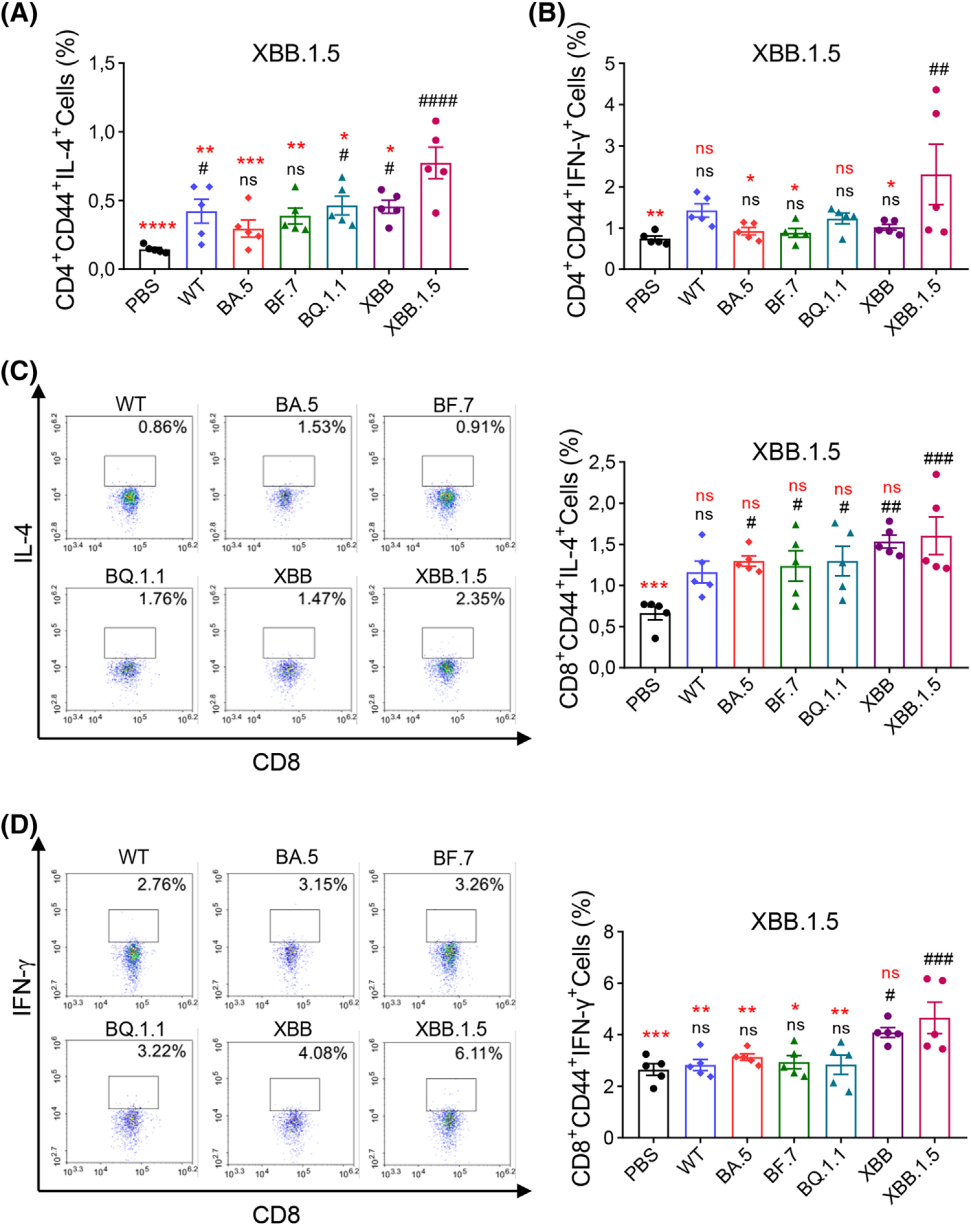
Splenic T‐cell responses against receptor‐binding domain (RBD)‐XBB.1.5 in immunized mice. Mice (*n* = 5/group) were intramuscularly immunized with PBS, spike‐WT, spike‐Omicron BQ.1.1, spike‐Omicron BA.5, spike‐Omicron XBB, spike‐Omicron BF.7, or spike‐Omicron XBB.1.5 on days 0, 21, and 42 intramuscularly. On day 56, splenic lymphocytes were isolated and stimulated with RBD‐XBB.1.5 antigens for 48 h and analyzed with flow cytometry. The frequencies of CD4^+^CD44^+^IL‐4^+^ (A), CD4^+^CD44^+^IFN‐γ^+^ (B), CD8^+^CD44^+^IL‐4^+^ (C), and CD8^+^CD44^+^IFN‐γ^+^ (D) T cells. (C and D) Representative flow cytometry plots (left) and quantification (right) of interleukin‐4 (IL‐4) (C) and interferon‐γ (IFN‐γ) (D) expression on CD8^+^CD44^+^ T cells (*n* = 5). Data are presented as mean ± SEM. ^*^ *p*‐Values compared with the Spike‐XBB.1.5 group. ^#^ *p*‐Values compared with the PBS group. ^*/#^ *p* < 0.05, ^**/##^ *p* < 0.01, ^***/###^ *p* < 0.001, ^****/####^ *p* < 0.0001. Black ns represents comparison with the PBS group. Red ns represents comparison with the Spike‐XBB.1.5 group.

Cellular immunity is also a crucial component of adaptive immunity for reducing disease severity and controlling pathogen infection, which involves both CD4^+^ and CD8^+^ T‐cell responses.^22^ The establishment of helper T‐cell (CD4^+^) responses is required for the formation of strong antibody responses as well as cytotoxic CD8^+^ T‐cell responses.^30^ Both antigen‐specific CD4^+^ and CD8^+^ T‐cell immune responses have been detected after SARS‐CoV‐2 infection or vaccination, which is closely related to the activation of humoral immunity.^31^ Hence, we next evaluated the capacities of the six vaccines in activating cellular immune responses. Two weeks after the third vaccination, splenic lymphocytes of the immunized mice were isolated and restimulated ex vivo with RBD‐XBB.1.5 or RBD‐BA.5 antigens for 48 h. T‐cell responses were then determined with flow cytometry and intracellular cytokine staining. After RBD‐XBB.1.5 antigen restimulation, immunization with spike‐WT, spike‐BQ.1.1, spike‐XBB, and spike‐XBB.1.5 induced a higher proportion of interleukin‐4 (IL‐4)‐producing antigen‐experienced (CD44^+^) CD4^+^ T cells than immunization with PBS, suggesting the activation of Th2‐biased responses against XBB.1.5 (Figure 5A).^32^, ^33^ No significant changes in the generation of IL‐4 were observed in the spike‐BA.5 and spike‐BF.7‐immunized mice. Among all the tested vaccines, only the spike‐XBB.1.5 vaccine increased the production of interferon‐γ (IFN‐γ) (Th1‐biased) in CD4^+^ T cells exposed to RBD‐XBB.1.5 antigen (Figure 5B). Therefore, splenocyte cytokine release profiles confirmed both Th1 and Th2 biases in spike‐XBB.1.5 vaccinated mice.

We next investigated the impact of the spike vaccines on cytotoxic T cells. When stimulated with RBD‐XBB.1.5, antigen‐experienced CD8^+^ T cells in the spike‐BA.5, spike‐BF.7, spike‐BQ.1.1, spike‐XBB, and spike‐XBB.1.5 groups produced higher levels of IL‐4 than those in the PBS group (Figure 5C). However, only immunization with spike‐XBB and spike‐XBB.1.5 enhanced the production of IFN‐γ in antigen‐specific CD8^+^ T cells (Figure 5D), which is a typical marker for cytotoxic T cells that are responsible for eradicating infected cells and controlling virus infection.^34^

**FIGURE 6:**
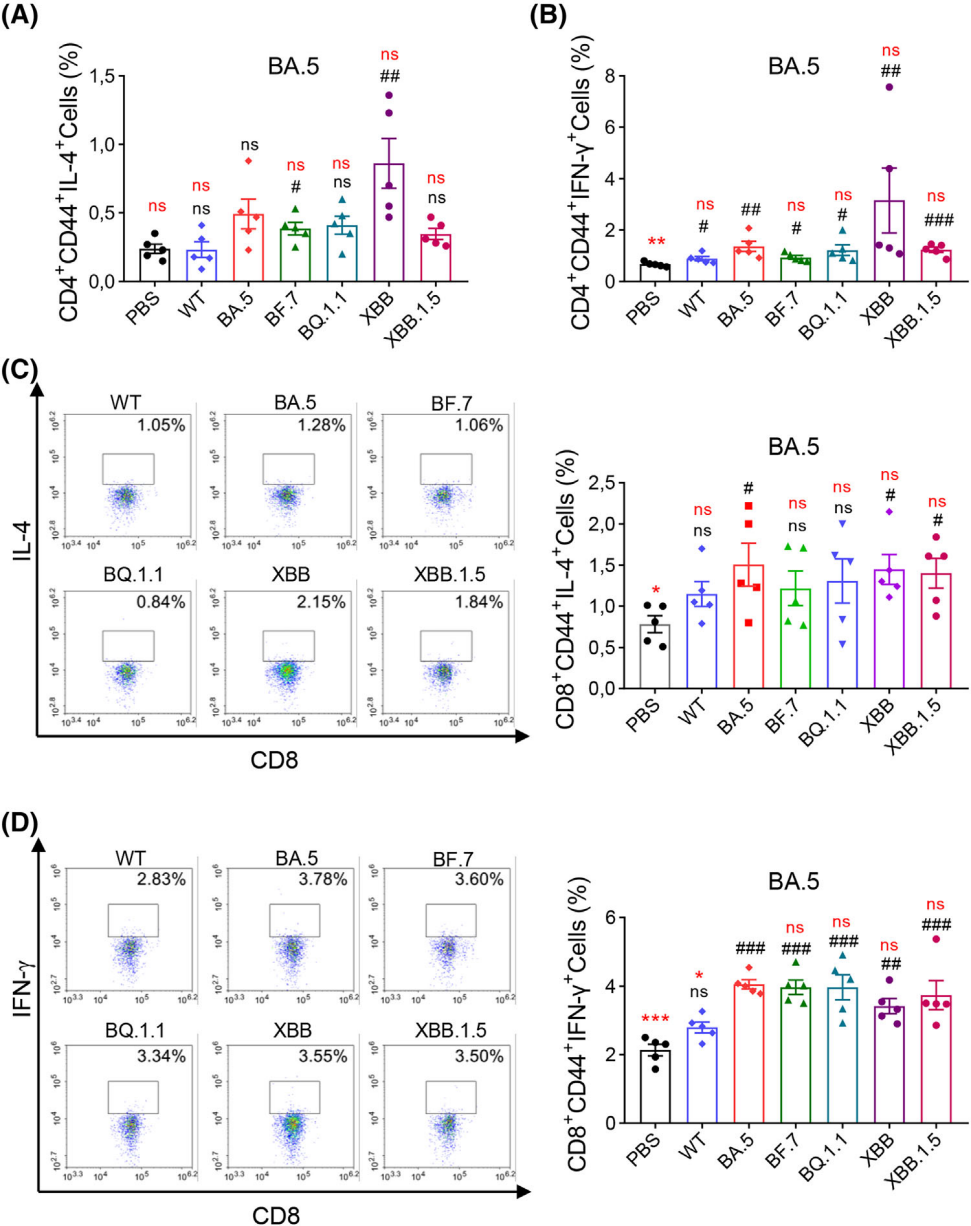
Splenic T‐cell responses against receptor‐binding domain (RBD)‐BA.5 in immunized mice. On day 56, splenic lymphocytes from immunized mice were isolated and stimulated with RBD‐BA.5 antigens for 48 h and analyzed with flow cytometry. The frequencies of CD4^+^CD44^+^IL‐4^+^ (A), CD4^+^CD44^+^IFN‐γ^+^ (B), CD8^+^CD44^+^IL‐4^+^ (C), and CD8^+^CD44^+^IFN‐γ^+^ (D) T cells. (C and D) Representative flow cytometry plots (left) and quantification (right) of interleukin‐4 (IL‐4) (C) and interferon‐γ (IFN‐γ) (D) expression on CD8^+^CD44^+^ T cells (*n* = 5). Data are presented as mean ± SEM. ^*^ *p*‐Values compared with the Spike‐BA.5 group. ^#^ *p*‐Values compared with the PBS group. ^*/#^ *p* < 0.05, ^**/##^ *p* < 0.01, ^***/###^ *p* < 0.001, ^****/####^ *p* < 0.0001. Black ns represents comparison with the PBS group. Red ns represents comparison with the Spike‐BA.5 group.

We also restimulated splenic T cells with RBD‐BA.5 proteins to evaluate the cellular immunity against the BA.5 subvariant. The results indicated that only spike‐BF.7 and spike‐XBB vaccines induced Th2‐biased immune responses (IL‐4, Figure 6A), whereas all of the six vaccines effectively induced Th2‐biased immunity (IFN‐γ, Figure 6B) against the BA.5 strain. No significant changes were observed in the frequencies of IFN‐γ‐producing CD4^+^CD44^+^ T cells in mice immunized with different spike proteins. In addition, vaccination with spike‐BA.5, spike‐XBB, and spike‐XBB.1.5 promoted the generation of IL‐4 in RBD‐BA.5‐restimulated CD8^+^CD44^+^ T cells in mice (Figure 6C). We also discovered a drastic increase in the frequency of cytotoxic CD8^+^CD44^+^ T cells in mice immunized with spike‐BA.5, spike‐BF.7, and spike‐BQ.1.1 against BA.5 subvariant (Figure 6D), suggesting the induction of cross‐protective cellular immunity between these Omicron variants similar to the results from the neutralizing assay (Figure 4B–D). Of note, immunization with spike‐XBB and spike‐XBB.1.5 also induced high proportions of cytotoxic T‐cell responses against BA.5 subvariants, which shows no inferiority over spike‐BA.5 vaccine immunization (Figure 6D). Taken together, these results demonstrated that the cellular immunity induced by spike‐BA.5, spike‐BF.7, and spike‐BQ.1.1 is primarily responsive to BA.5, which showed limited efficacy against the XBB.1.5 subvariant. In contrast, the spike‐XBB.1.5 vaccine can activate protective T‐cell responses against both BA.5 and XBB.1.5. Since spike‐XBB.1.5 contains the most RBD mutations, we speculated that it can induce broad cellular immunity against other earlier Omicron subvariants, which merits further exploration. In summary, these results further supported the broad protectivity of the spike‐XBB.1.5 vaccine against Omicron variants.

## 3 DISCUSSION

Different from other VOCs, Omicron variants have evolved with considerable diversity and are now categorized into five lineages based on the chronological order of appearance, including BA.1, BA.2, BA.3, BA.4, and BA.5.^5^ Since September 2022, BA.5 has caused a new global wave of infection, which is still one of the dominating strains in some countries such as China.^5^, ^24^, ^35^ Omicron variants are featured by high mutation rate, high transmissibility, and immune evasion.^2^ What's more, a complex connection was observed among different Omicron variants. Intra‐VOC recombination events are very common, creating upgraded generations of Omicron sublineages. A typical example is the combination or fusion of two BA.2 sublineages: BA.2.10.1 and BA.2.75, which created the XBB sublineage with increased resistance to host immunity but hampered ability to infect host cells.^35^, ^36^ XBB.1.5 has further evolved from XBB with higher affinity to host cells while continuing to evade the bodies’ antiviral immunity, making it the most contagious variant of SARS‐CoV‐2 so far.^35^ Since its first discovery in late 2022, it has overtaken other circulating strains and become dominant in the USA in such a short time. It is likely that XBB.1.5 will be the cause of the next global wave of COVID‐19 in the foreseeable future.

Current data indicate that XBB.1.5 strain is highly resistant to monoclonal antibodies and convalescent plasma treatment.^8^, ^10^ As more people will be infected with XBB.1.5, the absolute numbers of severe cases and deaths will certainly increase. It is predictable that the present treatment options will be poor in controlling XBB.1.5 infection. Therefore, a vaccine that could induce universally protective immunity against various Omicron subvariants, especially the XBB.1.5 strain, could minimize the harm they bring to people at the lowest cost. In the present study, we explored the efficacy of a brunch of vaccines designed based on the spike proteins of the WT SARS‐CoV‐2 (spike‐WT) or various Omicron subvariants, including spike‐BA.5, spike‐BF.7, spike‐BQ.1.1, spike‐XBB, and spike‐XBB.1.5. After intramuscular immunization, all the vaccines induced high levels of antigen‐specific IgG antibodies in the serum in mice (Figure 2). We also evaluated the functions of antibodies with a pseudovirus‐neutralizing assay. Strikingly, we discovered that of all the tested spike protein vaccines, spike‐XBB.1.5 protein induced the highest neutralizing capacity against XBB and XBB.1.5 pseudoviruses. It also showed remarkable protectivity against the BA.5, and BF.7 strains. As for BQ.1 and BQ.1.1 subvariants, the protective effect of spike‐XBB.1.15 vaccine showed a slight decrease (5.8–7.2‐fold) compared to the XBB.1.5 strain, while the 50% neutralizing titers still remained relatively high at around 10^3^ (Figure 3A). In contrast, spike‐BA.5, spike‐BF.7, and spike‐BQ.1.1 vaccines can only effectively block the infection of BA.5, BF.7, BQ.1, and BQ.1.1 Omicron strains. Their efficacy against the most contagious XBB.1.5 subvariant was significantly low (Figure 4B–D). These results indicated that spike‐XBB.1.5 induced broad‐spectrum humoral immunity against XBB‐1.5‐included Omicron variants.

In this study, we innovatively investigated the relationship between the number of mutations in RBD and the humoral immunity induced by various vaccines with correlation analysis. We surprisingly discovered that the protective effect of the vaccines against Omicron subvariants with fewer RBD mutations is only slightly reduced, unchanged, or even increased. For instance, the sera from mice immunized with spike‐BF.7 and spike‐BQ.1.1 showed stronger neutralization of the BA.5 than their targeted strains (Figure 4C,D). We thought that is because spike‐BF.7 and spike‐BQ.1.1 vaccines contain more mutations in their RBD than that in the RBD of BA.5 strain (Figure 1B), which makes the induced antibodies capable of recognizing and binding to the subvariants with fewer mutations in the RBD. In the case of spike‐XBB and spike‐XBB.1.5 vaccines, their efficacy in neutralizing earlier Omicron subvariants was slightly reduced (Figure 3A,B), which we believed to be a result of some unique mutations in the earlier strains that cannot be found in the structure of RBD in XBB and XBB.1.5, for example, the G339D, L452R, and F486V mutations. However, the efficacy of these vaccines in protecting against viruses that possess more mutations in the RBD is severely compromised and the NAb titers reduced as the number of RBD mutations increased, which showed a strong negative correlation (Figure 4B–D). Therefore, the mutations in RBD of SARS‐CoV‐2 significantly interfered with the epitopes of these Omicron vaccines, leading to altered antigenicity and immunogenicity. As for viruses, the accumulation of RBD mutations caused enhanced resistance to humoral immunity (Figure 4), allowing the highly mutated subvariants to evade existing immunity and cause breakthrough infection. This may be because these mutation sites are closely associated with RBD and antibody binding, which can be further proven by the data from spike‐WT vaccine and WT virus neutralization. The spike‐WT vaccine produced high protective antibodies against the WT strain, but the protection against other Omicron sublineages decreased with the increase in the number of mutations in their RBD. On the contrary, in a variety of Omicron‐targeting vaccines, NAb titers against WT pseudovirus declined sharply and showed a gradual decline with the accumulation of vaccines’ RBD mutations, from 460 in spike‐BA.5 to 24 in spike‐XBB‐immunized mice. Thus, a vaccine designed to contain more mutations in RBD can resist the increased immune escape caused by the accumulation of virus mutations, thus forming a strong protective immunity against multiple Omicron sublineages. This makes our spike‐XBB.1.5 vaccine ideal for the current Omicron epidemic. However, we also noticed some slightly abnormal results in this study. We found that spike‐BQ.1.1 and spike‐XBB.1.5 vaccination induced higher NAb titers against WT pseudovirus than spike‐BF.7 and spike‐XBB vaccines, respectively, the formers of which possess more mutations in RBD and were supposed to be less effective against WT SARS‐CoV‐2. We thought this may be caused by mutations in the spike protein outside the RBD structure, which will be further studied in our future work.

Cellular immunity is also an essential part of antiviral immunity. Upon antigen exposure, antigen‐presenting cells can uptake, process, and present antigens to T cells, activating CD4^+^ and CD8^+^ T cells. CD4^+^ T helper cells are crucial for the establishment of humoral immune responses and cytotoxic CD8^+^ T‐cell immunity, the latter of which is responsible for clearing host cells infected by virus. It has been reported that CD8^+^ and CD4^+^ T cells were important components of the antiviral immunity against SARS‐CoV‐2.^37^ In the current study, we found that immunization with spike‐XBB.1.5 induced strong T‐cell responses against both RBD‐XBB.1.5 and RBD‐BA.5 antigens, indicating its potential in activating universal cellular immunity against Omicron. In contrast, other vaccines that target earlier Omicron strains failed to induce cytotoxic CD8^+^ T‐cell response against XBB.1.5. These results suggested that XBB.1.5 subvariant can escape from both pre‐existing humoral and cellular immunity, which can be salvaged by the spike‐XBB.1.5 protein vaccine.

Taken together, our results indicated that among all the tested Omicron vaccines, the spike‐XBB.1.5 protein vaccine induced strong protective immune responses against most of the circulating Omicron sublineages, including the most concerned XBB.1.5 strain at present. A strong negative correlation was observed between NAb titers and the number of mutations in RBD of Omicron sublineages in multiple vaccine models. Meanwhile, we found that vaccines targeting Omicron variants with more RBD mutations showed strong efficacy in neutralizing those with fewer mutations in RBD, making spike‐XBB.1.5, which has the most RBD mutations, a promising vaccine candidate against the existing and future SARS‐CoV‐2 Omicron variants.

## 4 MATERIALS AND METHODS

### 4.1 Cell lines and cell culture

Human embryonic kidney 293T (HEK293T) cells were obtained from the American Type Culture Collection. ACE2‐expressing HEK293T cells (293T/ACE2) were constructed by our team as discussed in previous reports.^21^ Cells were grown and maintained in Dulbecco's modified Eagle's medium (Gibco, USA) containing fetal bovine serum (10%, FBS), streptomycin (100 µg/mL), and penicillin (100 U/mL) in a 37°C incubator with 5% CO_2_.

### 4.2 Vaccine formulations

The recombinant SARS‐CoV‐2 spike proteins, including spike‐WT, spike‐BA.5, spike‐BF.7, spike‐BQ.1.1, spike‐XBB, and spike‐XBB.1.5 were expressed with HEK293T cells, which have His tag at the C‐terminus. The spike proteins contain AA Val 16‐Pro 1213 and have a calculated molecular weight (MW) of 137.7–137.8 kDa. Substitutions of proline (F817P, A892P, A899P, A942P, K986P, V987P) are introduced to stabilize the trimeric prefusion state of spike proteins and mutations in alanine (R683A and R685A) are involved in removing the furin cleavage site. The purity of the proteins was over 95%. Limulus amebocyte lysate (LAL) assay results indicated that the endotoxin levels of these proteins were no more than 1.0 EU/µg protein. The vaccines were prepared by mixing the spike proteins and the MF59‐like adjuvant at a volume ratio of 1:1. All of the recombinant proteins were provided by ACRO Biosystems.

### 4.3 Vaccination of mice

Six‐ to eight‐week‐old female NIH mice were purchased from Vital River (Beijing, China) and bred in a specific pathogen‐free room with stable temperature and humidity. The mice were randomized into seven groups (six mice per group) and immunized with spike‐WT, spike‐BA.5, spike‐BF.7, spike‐BQ.1.1, spike‐XBB, or spike‐XBB.1.5 in the presence of MF59‐like adjuvant on days 0, 21, and 42 via intramuscular injection. Mice immunized with PBS were used as control. Every mouse was dosed with 10 µg spike proteins with MF59‐like adjuvant according to our previous research.^21^, ^22^ Blood samples and spleen were collected on day 56 to detect humoral and cellular immune responses, respectively. All animal experiments have been approved by the Institutional Animal Care and Use Committee of Sichuan University (Sichuan, China). Immune sera were maintained at −20°C before use.

### 4.4 Enzyme‐linked immunosorbent assays

To determine antigen‐specific IgG titers, recombinant spike‐WT, spike‐BA.5, spike‐BF.7, spike‐BQ.1.1, spike‐XBB, or spike‐XBB.1.5 proteins were used to coat 96‐well plates (NUNCMaxiSorp, Thermo Fisher Scientific) in carbonate coating buffer (50 mM, pH 9.6) at 1 µg/mL at 4°C overnight. After three washes with PBS containing 0.1% Tween‐20 (PBST), the precoated plates were blocked with 200 µL/well of 1% bovine serum albumin for 1 h at room temperature. The immune sera were thawed slowly at 4°C, serially diluted, added to the plates, and incubated at 37°C for 1 h. After another three washes with PBST, horseradish peroxidase‐conjugated anti‐mouse IgG antibodies (Invitrogen, 1:10,000) were added to the plates (100 µL/well) and incubated for 1 h at 37°C. Then, the plates were washed five times before the addition of 3,3′,5,5′‐tetramethyl biphenyl diamine (100 µL/well) for color development. After 10–15 min, 100 µL/well of ELISA stop solution (Beyotime, China) was added to stop the reaction. Finally, a microplate reader (Spectramax ABS, Molecular Devices) with SoftMax Pro 7.1 software was applied to read the absorbance value.

### 4.5 Pseudovirus neutralization assay

Pseudoviruses of various Omicron subvariants were used to evaluate the neutralizing capacities of the immune sera in mice immunized with recombinant spike protein vaccines. The luciferase‐expressing pseudoviruses of WT, BA.5, BF.7, BQ.1, BQ.1.1, XBB, and XBB.1.5 strains were purchased from Genomeditech (China, Shanghai). In brief, immune sera from the immunized mice were serially diluted and incubated with the pseudoviruses for 1 h at 37°C. Next, the mixtures of immune sera and pseudoviruses were added to 293T/ACE2 cells in 96‐well dark plates (WHB, 1.2 × 10^4^ cell/well) and incubated for 48 h in the cell culturing incubator to allow the expression of luciferase. Eventually, cell supernatants were replaced with 100 µL lysis reagent with luciferase substrate in a luciferase kit (Promega, USA). The relative light unit was measured with a multi‐mode microplate reader (PerkinElmer, USA) with Kaleido 3.0 software.

### 4.6 Isolation of splenic lymphocytes

On day 56, the immunized mice were sacrificed and the spleen was harvested and minced into tiny pieces. Splenic lymphocytes were isolated with mouse lymphocyte separation medium (Dakewe Biotech Co., Ltd., China), treated with red blood cell lysis buffer, and resuspended with RPMI 1640 culture medium (Gibco, USA) containing RBD‐XBB.1.5 or RBD‐BA.5 antigens (10 µg/mL), 10% FBS, streptomycin (100 µg/mL), penicillin (100 U/mL), sodium pyruvate (1 mM), IL‐2 (500 U/mL), and 50 mM b‐mercaptoethanol. After 44 h culturing, brefeldin A (BioLegend) was added for 4 h before flow cytometry assessment.

### 4.7 Assessment of T‐cell responses in spleen

Flow cytometry assays were performed with a NovoCyte Flow Cytometer (ACEA Biosciences, Inc.). The results were analyzed with NovoExpress software (1.4.1).

The restimulated lymphocytes were collected and stained with the following antibodies (BioLegend) for 30 min at 4°C: PerCP/Cyanine5.5‐conjugated anti‐mouse CD3, FITC‐conjugated anti‐mouse CD4, APC‐conjugated anti‐mouse CD8, and Brilliant Violet 510‐conjugated anti‐mouse CD44. For intracellular cytokine staining, the stained lymphocytes were fixed and permeabilized with the Fixation and Permeabilization kit (BD Biosciences, USA) according to the manufacturer's instructions. Then, Brilliant Violet 421‐conjugated anti‐mouse IL‐4 and PE‐conjugated anti‐mouse IFN‐γ antibodies were added and incubated for 1 h at room temperature or overnight at 4°C. Cells were then washed with PBS once before flow cytometry analysis.

### 4.8 Statistical analysis

Statistical analyses were carried out with Prism software (GraphPad Prism 8.0). Unpaired Student's *t*‐test was used to compare two groups, while comparisons among three or more groups involved one‐way ANOVA followed by Tukey's multiple comparison post hoc test. *p* < 0.05 was considered significant.

## AUTHOR CONTRIBUTIONS

X.W. provided the research concepts and designed the experiments. X.S. and J.L. prepared MF59‐like adjuvant. C.H. performed animal immunization, ELISA, and pseudovirus neutralization assays. C.H., H.L., A.A., J.Y., and W.H. performed flow cytometry. G.S., W.W., and L.Y. were involved with data acquisition. A.A. and C.H. drafted the manuscript. X.W. revised and edited the manuscript. All authors have read and approved the final manuscript.

## CONFLICT OF INTEREST STATEMENT

This work was supported by the WestVac Biopharma Co. Ltd. Jiong Li, Wei Wang, Li Yang, Guobo Shen and Xiawei Wei are employees of WestVac Biopharma Co. Ltd. Remaining authors declare no conflicts of interest.

## ETHICS STATEMENT

All animal experiments were approved by the Institutional Animal Care and Use Committee of Sichuan University (Chengdu, Sichuan, China).

## Data Availability

The data included in this study are available upon request from the corresponding author.
